# Long-Term Outcomes of Transanal Irrigation for Bowel Dysfunction

**DOI:** 10.7759/cureus.42507

**Published:** 2023-07-26

**Authors:** Panagiotis Tamvakeras, Clare Horrobin, Jessica Chang, Mark Chapman

**Affiliations:** 1 Colorectal Surgery, University Hospitals Birmingham NHS Foundation Trust, The Royal Town of Sutton Coldfield, GBR; 2 Lower GI Physiology, University Hospitals Birmingham NHS Foundation Trust, The Royal Town of Sutton Coldfield, GBR

**Keywords:** pelvic floor dysfunction, transanal irrigation, rectal irrigation, bowel dysfunction, pelvic floor disorder

## Abstract

Introduction: Transanal irrigation (TAI) improves bowel function and quality of life in patients with neurogenic bowel disease compared to conservative bowel care. Its use has been extended to a range of defecatory disorders. However, data on long-term benefits and compliance are lacking. We aim to evaluate the long-term efficacy of TAI by examining compliance and patient outcomes over a five-year period.

Methods: This study is a five-year retrospective review of patients practising TAI in a district general hospital. Patient demographics, indications, long-term compliance, adverse events, and patient-reported Qufora bowel symptom bother scores were analysed.

Results: A cohort of 18 patients had a median age of 61 (range 23-91) and were predominantly female (83.5%). The reasons for bowel dysfunction were diverse: low anterior resection syndrome, neurogenic bowel, congenital anorectal malformations, obstructed defecation, and functional disorders. Predominant symptoms were constipation (9), faecal incontinence (7), and mixed (2). Both high-volume (catheter and cone) and low-volume (mini cone) irrigation devices were used. Fourteen patients continued regular irrigation at a median follow-up of 27.7 months (range 5.1-72.3), while four had discontinued at a median follow-up of 4.8 months. The reasons for discontinuation were inadequate rectal evacuation and spontaneous improvement of symptoms. In the compliant group, there was a significant improvement in bowel symptom scores (p=0.003). No major adverse events, such as significant rectal bleeding or perforation, were noted.

Conclusion: In this small cohort, TAI was safe and effective for long-term use; however, a fifth of patients discontinued treatment. Further work needs to be done to identify those patients who will benefit from TAI.

## Introduction

Transanal irrigation (TAI) is the process by which water is introduced to the rectum through the anus in order to assist with the evacuation of faeces in patients with bowel dysfunction. A randomised controlled trial demonstrated that TAI improves faecal incontinence, constipation, and quality of life in patients with neurogenic bowel dysfunction compared to conservative bowel care [1]. Regular irrigation can offer a degree of control over the time and place of bowel movements. Evacuation of the rectosigmoid through irrigation can prevent over-accumulation of faeces and accelerate transit through the rest of the colon in patients with chronic constipation, while in those with faecal incontinence, it can prevent leakage between irrigations for an average of two days [2].

TAI is a minimally invasive adjunct in the armamentarium of treatment options for bowel dysfunction. Importantly, it may help patients manage their symptoms when other conservative measures fail without the need for surgical interventions such as antegrade colonic irrigation, sacral neuromodulation, and stoma formation [3].

Although TAI is most commonly utilised in the management of neurogenic bowel dysfunction, its use has been expanded to include other conditions affecting defecation, such as low anterior resection syndrome, obstructed defecation, chronic constipation, and faecal incontinence of heterogenous origin, including trauma and functional bowel disorders [4-6].

TAI can be time-consuming, psychologically distressing, and ineffective for many patients, and compliance is generally poor with high discontinuation rates [6,7]. Furthermore, assessing compliance can be challenging since long-term follow-up data on TAI patients are lacking in most but a few small studies [8-9]. The aim of this study was to evaluate the long-term efficacy of TAI by examining compliance over a long follow-up period and patient-reported satisfaction.

## Materials and methods

A retrospective case note review was undertaken for all patients who commenced TAI through the pelvic floor service at Good Hope Hospital, Sutton Coldfield, UK, between June 2016 and November 2021. Eighteen patients were identified, initially assessed in an outpatient clinic by a colorectal surgeon, and referred to the pelvic floor service. All patients referred for irrigation had failed first-line management with dietary/lifestyle modifications, laxatives, or antidiarrhoeal medications. Then, they had a face-to-face consultation with the specialist nurse to review their history and medications and discuss the different irrigation devices. Subsequently, an in-person teaching session was arranged in the Gastrointestinal Physiology unit with the device of choice, following which self-irrigation was commenced at home. Routine telephone follow-up was then arranged at 2, 6, and 12 weeks, 6 months, 1 year, and ad hoc thereafter, while patients were encouraged to contact the department directly if they had any concerns. Elderly patients or those experiencing ongoing issues received further telephone follow-up consultations every six months or yearly.

Patients were provided with the Qufora bowel progress diary (MacGregor Healthcare, Tranent, UK). They were asked to rate how bothersome their bowel symptoms were on a linear scale of the Qufora bowel symptom bother visual analogue score used in the diary, from 0=not at all to 10=all the time [10]. For most patients, a baseline measurement pre-irrigation was obtained at the initial teaching session, and post-irrigation scores were obtained at different times during the telephone follow-up consultations. The lowest post-irrigation score for each patient was used for the data analysis.

This study was approved by the University Hospitals Birmingham NHS Foundation Trust audit department and was conducted in accordance with the Helsinki Declaration. As a retrospective study, formal consent was not required.

Statistical analysis

All statistical analysis was computed using SPSS 28.0 (IBM Corp., Armonk, NY, USA). For the compliant group, the Wilcoxon signed-rank test was used to analyse the difference between pre-irrigation and post-irrigation scores. Significance was set at a p-value of 0.05.

## Results

Patient population

Our patient cohort had a median age of 61 (range 23-91), with a female predominance (83.5% vs. 16.5%). Constipation was the main symptom for half of the patients; seven predominantly had faecal incontinence, while two experienced balanced symptoms of incomplete evacuation and passive incontinence. Underlying pathology was diverse, with the most common diagnoses being low anterior resection syndrome in four (22%), and congenital anorectal malformations in four patients (22%), which included two cases of imperforate anus with pull-through procedures in infancy, one case of ectopic anus that had undergone posterior sagittal anorectoplasty, and a patient with sacral agenesis. Obstructive defecation and neurogenic bowel syndrome, which included cases of spina bifida and cauda equina, were the second most common indications. Other diagnoses included functional bowel disorder, anal stenosis, and one case of persistent rectal stump leakage following Hartmann’s procedure (Table 1).

**Table 1:** Patient characteristics

Population characteristics (n=18)	Number of patients/percentage
Sex	
Male	3/16.5%
Female	15/83.5%
Predominant symptoms	
Constipation	9/50%
Faecal incontinence	7/39%
Mixed symptoms	2/11%
Underlying diagnosis	
Low anterior resection syndrome	4/22%
Obstructive defecation	3/16.5%
Congenital anorectal malformations	4/22%
Sacral agenesis	1/5.5%
​​​​​​​Ectopic anus	1/5.5%
​​​​​​​Imperforate anus	2/11%
Neurogenic bowel dysfunction	3/16.5%
​​​​​​​Cauda equina syndrome	2/11%
​​​​​​​Spina bifida	1/5.5%
Diversion colitis	1/5.5%
Anal stenosis	1/5.5%
Functional bowel disorder	2/11%

Furthermore, there was also a wide range of irrigation devices used at the assessment and teaching sessions prior to the commencement of transanal irrigation, focused on tailoring the device to suit the specific needs and preferences of each patient. High-volume irrigation systems were used by 66% of patients, with the Qufora IrriSedo Cone System (MacGregor Healthcare) being the most popular, while a third of the patients used low-volume irrigation systems (Table 2).

**Table 2:** Devices

Devices	Number of patients/percentage
Qufora IrriSedo Mini GO	1/5.5%
Qufora IrriSedo Mini Cone	2/11%
Qufora IrriSedo Cone System	6/33%
Peristeen	4/22%
Qufora IrriSedo Klick	2/11%
Qufora Irrisedo Mini	3/16.5%

Of the 18 patients that started transanal self-irrigation, 14 (78%) were continuing regularly at a median follow-up of 27.7 months (range 5.1-72.3), while 4 (22%) had stopped.

Non-adopters of transanal irrigation

Four patients discontinued rectal irrigation, representing 22% of the study population. All of them were female. One patient with faecal incontinence following spinal decompression surgery discontinued irrigation at 14 months as her sphincter function and perineal sensation recovered almost fully and irrigation was no longer deemed necessary. The other three patients were treated for predominantly constipation and all stopped due to the ineffectiveness of irrigation to stimulate evacuation and, in one instance, abdominal colicky pain during use. One patient proceeded to have a colostomy. The median length of irrigation use in this group was 4.8 months (range, 3.1-14 months).

Adopters of transanal irrigation

The remaining 14 (78%) patients tolerated irrigation well and reported some benefits. The most common irrigation frequency was once every alternate day. Reported issues included occasional anal pain (21%), incomplete evacuation (14%), inefficiency in preventing passive leakage (14%), and anxiety having an adverse impact on mental health (5.5%). One patient passed away six months after her last follow-up following a new diagnosis of advanced gynaecological malignancy, and her death was unrelated to rectal irrigation. There were no instances of rectal bleeding or perforation. The median follow-up in this group was 27.7 months (range, 5.1-72.3 months). Patient-reported bowel symptom bother scores before and after the commencement of rectal irrigation were available for 13 patients. A Wilcoxon signed rank test revealed that scores were significantly lower after TAI (median = 5, n = 13) compared to before (median = 9.5, n = 13), z = −2.96, p = 0.003, with a large effect size of r = 0.58.

## Discussion

Our follow-up of 27.7 months is among the longest in the literature. Hamonet-Torny et al. reported a mean follow-up of 31 months for 16 patients with neurogenic bowel disorders irrigating with Peristeen with a 62.5% continuation rate [8]. The longest published mean follow-up is 40 months in a population of 49 patients with multiple sclerosis also irrigating with Peristeen, with a 55% rate of continuation [9].

Poor tolerance of rectal irrigation has been described, but discontinuation rates range widely between studies of varying sample sizes, indications, and follow-up durations. Juul and Christensen described a discontinuation rate of 34% at 12 months in their large cohort of 507 patients with heterogeneous indications [11]. Other studies with over 100 patients reported discontinuation rates of 47-57% [6,12]. In our study, only 22% of patients stopped using irrigation, which compares favourably to the literature. This outcome may reflect the quality of training that the patients received, the regular follow-up, and the ongoing support. Furthermore, unlike many previous studies that used a single irrigation device, our patient cohort could choose from a variety of high- and low-volume devices with the guidance of the specialist nurse. This tailored approach may have contributed to improved compliance.

The literature reveals a wide variation in the tolerance and benefit of rectal irrigation among patients, indicating the need for improving patient selection. Few studies report on anorectal physiology parameters without consistent findings. Christensen et al. found low rectal volume at the urge to defecate and low maximal rectal capacity as predictors of successful treatment [13]. Those findings were not validated by other studies investigating anorectal physiology in the context of TAI, while Passanati et al. reported impaired anal electrosensitivity as a predictive factor of response to treatment [9,12,14]. Other factors relating to successful outcomes, such as demographics, underlying bowel pathology, and various clinical factors, have been studied, but none has consistently been associated with efficacy and compliance [7,8]. The success of the first training session has been identified as an independent predictor of compliance with irrigation [12]. That is in keeping with our experience of the importance of patient education and support for successful treatment. Furthermore, research for predictive factors that will improve patient selection may need to focus on parameters such as mental health, socioeconomic status, and personality types.

Various validated patient-reported outcome measures (PROMs) have been used to describe the impact of TAI on bowel function, such as the Neurogenic Bowel Dysfunction Score, the Cleveland Clinic Constipation Score, and the St. Marks Incontinence Score [8,11,15]. Additionally, the effect of irrigation on quality of life has been reported with validated, disease-specific PROMs such as the Patient Assessment of Constipation Quality of Life Score (PAC-QoL) and the Faecal Incontinence Quality of Life Scale (FI-QoL) [16]. Although these PROMs are useful at describing the patient’s perspectives on the severity of their symptoms in a homogenous population, they offer limited comparability for diverse patient populations.

This retrospective study showed that transanal irrigation significantly improved bowel symptom bother scores (p=0.003). The bowel symptom bother score is a simple patient-reported visual analogue score of overall satisfaction with bowel function, focusing on the level of impairment rather than the severity of symptoms [10]. This was applicable to our patient cohort as a whole, which was diverse in terms of underlying diagnoses and predominant symptoms. Furthermore, rectal irrigation requires a significant investment in time and effort, and patients tend to discontinue ineffective treatments early, as supported by the median time to discontinuation of 4.8 months in our study. It is of note that the duration of treatment alone has been considered a surrogate marker of efficacy [6].

TAI has also been shown to be cost-effective compared to standard bowel care in patients with neurogenic bowel dysfunction in a study designed for the NHS [17]. Similar analyses have also confirmed that TAI is cost-effective in Germany and Japan [18,19]. The gains for the healthcare system seem to stem from fewer stoma operations and hospital admissions, fewer urinary tract infections, and less nursing support at home for patients self-administering irrigation compared to standard bowel care [17,18].

This study is limited by its retrospective design, which resulted in incomplete data regarding the pre-treatment and post-treatment bowel symptom bother scores. Furthermore, it is a single-institution study with a small and heterogeneous cohort, which may limit the generalisation of the results to other populations and settings.

## Conclusions

Transanal irrigation is a useful and cost-effective adjunct in the management of patients with intractable defecatory problems. However, it seems to have limited effectiveness for a significant number of patients who tend to discontinue treatment early. The patient- and disease-related predictive factors for a successful outcome remain unknown. Further research into identifying the group of patients with bowel dysfunction who will benefit most from this treatment could improve the patient experience and the allocation of healthcare resources.
